# Co-Inhibition of P-gp and Hsp90 by an Isatin-Derived Compound Contributes to the Increase of the Chemosensitivity of MCF7/ADR-Resistant Cells to Doxorubicin

**DOI:** 10.3390/molecules27010090

**Published:** 2021-12-24

**Authors:** Ashraf N. Abdalla, Miriana Di Stefano, Giulio Poli, Tiziano Tuccinardi, Ammar Bader, Antonio Vassallo, Mohamed E. Abdallah, Mahmoud Zaki El-Readi, Bassem Refaat, Alanood S. Algarni, Rizwan Ahmad, Hamad M. Alkahtani, Alaa A.-M. Abdel-Aziz, Adel S. El-Azab, Aljawharah Alqathama

**Affiliations:** 1 College of Pharmacy, Umm Al-Qura University, Makkah 21955, Saudi Arabia; ambader@uqu.edu.sa (A.B.); aagarni@uqu.edu.sa (A.S.A.); aaqathama@uqu.edu.sa (A.A.); 2 Medicinal and Aromatic Plants Research Institute, National Center for Research, Khartoum 2404, Sudan; 3 Department of Pharmacy, University of Pisa, 56126 Pisa, Italy; miriana.distefano@phd.unipi.it (M.D.S.); giulio.poli@unipi.it (G.P.); tiziano.tuccinardi@unipi.it (T.T.); 4 Dipartimento di Scienze, Università Degli Studi della Basilicata, 85100 Potenza, Italy; antonio.vassallo@unibas.it; 5 Department of Clinical Biochemistry, Faculty of Medicine, Umm Al-Qura University, Makkah 21955, Saudi Arabia; mezubier@uqu.edu.sa (M.E.A.); mzreadi@uqu.edu.sa (M.Z.E.-R.); 6 Department of Biochemistry, Faculty of Pharmacy, Al-Azhar University, Assiut 71524, Egypt; 7 Department of Laboratory Medicine, Faculty of Applied Medical Sciences, Umm Al-Qura University, Makkah 21955, Saudi Arabia; barefaat@uqu.edu.sa; 8 Natural Products and Alternative Medicines, College of Clinical Pharmacy, Imam Abdulrahman Bin Faisal University, Dammam 31441, Saudi Arabia; rareiyadh@iau.edu.sa; 9 College of Pharmacy, King Saud University, Riyadh 11451, Saudi Arabia; ahamad@ksu.edu.sa (H.M.A.); almoenes@ksu.edu.sa (A.A.-M.A.-A.); adelazab@ksu.edu.sa (A.S.E.-A.)

**Keywords:** MDR, isatin, synergism with doxorubicin, apoptosis, P-gp, Hsp90, SPR, molecular docking and dynamics

## Abstract

Breast cancer is a complex and multi-drug resistant (MDR) disease, which could result in the failure of many chemotherapeutic clinical agents. Discovering effective molecules from natural products or by derivatization from known compounds is the interest of many research studies. The first objective of the present study is to investigate the cytotoxic combinatorial, chemosensitizing, and apoptotic effects of an isatin derived compound (5,5-diphenylimidazolidine-2,4-dione conjugated with 5-substituted isatin, named HAA_2021_ in the present study) against breast cancer cells (MCF7) and breast cancer cells resistant to doxorubicin (MCF7/ADR) when combined with doxorubicin. The second objective is to investigate the binding mode of HAA_2021_ withP-glycoprotein (P-gp) and heat shock protein 90 (Hsp90), and to determine whether their co-inhibition by HAA_2021_ contribute to the increase of the chemosensitization of MCF7/ADR cells to doxorubicin. The combination of HAA_2021_, at non-toxic doses, with doxorubicin synergistically inhibited the proliferation while inducing significant apoptosis in MCF7 cells. Moreover, HAA_2021_ increased the chemosensitization of MCF7/ADR cells to doxorubicin, resulting in increased cytotoxicity/selectivity and apoptosis-inducing efficiency compared with the effect of doxorubicin or HAA_2021_ alone against MCF7/ADR cells. Molecular modeling showed that two molecules of HAA_2021_ bind to P-gp at the same time, causing P-gp inhibitory effect of the MDR efflux pump, and accumulation of Rhodamine-123 (Rho123) in MCF7/ADR cells. Furthermore, HAA_2021_ stably interacted with Hsp90α more efficiently compared with 17-*N*-allylamino-17-demethoxygeldanamycin (17-AAG), which was confirmed with the surface plasmon resonance (SPR) and molecular modeling studies. Additionally, HAA_2021_ showed multi-target effects via the inhibition of Hsp90 and nuclear factor kappa B (NF-𝜅B) proteins in MCF7 and MCF7/ADR cells. Results of real time-PCR also confirmed the synergistic co-inhibition of P-gp/Hsp90α genes in MCF7/ADR cells. Further pharmacokinetic and in vivo studies are warranted for HAA_2021_ to confirm its anticancer capabilities.

## 1. Introduction

Although multi-target drugs have less possibilities of causing drug-drug interactions as well as more patient compliance and more predictable pharmacokinetics compared with drug-combinations, their benefits versus drawbacks are still controversial regarding clinical anticancer therapy, which is mainly due to their possible off-target side effects. Yet, their benefits outweigh the drawbacks when they are the best or the only option for complex, multifactor or multi-drug resistance (MDR) diseases [1], such as breast cancer.

Resistance to apoptosis is one of the most important hallmarks of cancer, and the deregulation of apoptosis causes the dramatic failure of breast cancer chemotherapy. One of the main reasons is the breast cancer MDR [2], which arises from numerous mechanisms, such as enhanced drug efflux, increased DNA repair capacity, and genetic factors in various cell biological processes [3,4]. The most widely studied proteins involved in MDR are the membrane transporters, including the main member of the ATP-binding cassette (ABC) transporter family: P-glycoprotein (P-gp) [5,6,7], which is associated with cancer growth and proliferation in the downstream pathways [8]. The combination of therapeutic agents with effective chemosensitizers, including doxorubicin, is one of the solutions to the cancer MDR [9]. Another important reason for perturbation of apoptosis is the upregulation of Hsp90, which is a molecular chaperone that stabilizes many signalling proteins. Therefore, as an example of relevant pro-apoptotic pathways, the inhibition of Hsp90 blocks the activation of nuclear factor kappa B (NF-𝜅B), and inhibits the VEGFR2 pathway, all leading to apoptotic events of the cancer cell machinery [10,11]. As a result, the discovery of multi-target drugs could lead to the upregulation of apoptosis and other related clickable therapeutic pathways.

The search for new anticancer agents requires huge screening programs on medicinal and non-medicinal plants [12], as well as the investigation on marine algae and living organisms, such as sponges and corals [13]. To overcome this problematic searching strategy, which is both expensive and time consuming, a few lead molecules can be chemically modified in order to improve their activity and selectivity or to reduce their toxicity [14]. One of these important modifiable drug classes are the isatins, which are natural compounds isolated from many plants including *Couroupita guianesis* [15]. They were the subject of extensive molecular modifications to improve their anticancer activity [16,17,18,19,20,21]. The isatin (indole-2,3-dione) is an endogenous oxidized indole building block, able to form many heterocyclic molecules, and it is expressed in various mammalian tissues and body fluids. Compounds containing isatin exhibit a broad spectrum of potential pharmacological actions, including anti-inflammatory, anti-microbial, antiviral, and anticancer properties [22,23,24].

Previous anticancer research indicated that isatins could be used, not only for their cytotoxic effects (such as inducing apoptosis by activation of caspase 3/7), but also for their ability to reverse MDR protein activity [3,4,18,25]. Novel isatin derivatives of podophyllotoxin have previously shown a significant anticancer activity against several cancer MDR cell lines, such as K562/ADR cells [19]. In another study, the anticancer mechanisms of action for indole-2,3-diones were associated with their affinity for tyrosine kinase receptors (RTKs) and their tendency to inhibit extracellular signal-regulated kinases (ERK), vascular endothelium growth factors and receptors (EGFR and VEGFR2), heat shock proteins, and P-gp expression. Additionally, melosine isolated from *Melodinus cochinchinensis*, and bengacarboline, isolated from *Ascidian didemnum*, both caused significant inhibition of MCF7 cells [15]. Therefore, the development of isatin-derived anticancer drugs has become an active research area in the pharmaceutical industry. Accordingly, many drugs containing indole moiety have been approved by the US Food and Drug Administration (FDA) for clinical use as anticancer agents, including sunitinib, osimertinib, alictinib, and panobinostat [7,15,26].

**Figure 1:**
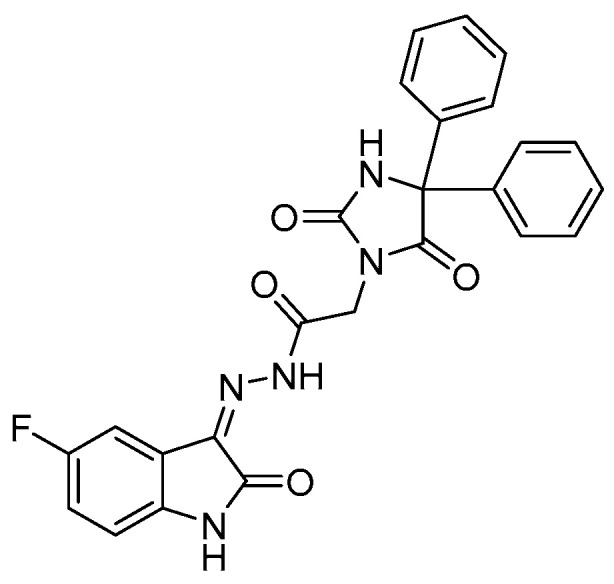
Molecular structure of the isatin derivative: Compound 16 [27], named HAA_2021_ in the present study.

One of our collaborating research groups in King Saud University, Saudi Arabia, previously synthesized 15 new heterocyclic isatin derivatives by hybridizing 5,5-diphenylimidazolidine-2,4-dione conjugated with 5-substituted isatin [27]. Those compounds have been tested for their cytotoxic activity in HeLa, A549, and MDA-MB-231 cells. Compound No. 16 (Figure 1) was the most potent candidate against the cell lines, and showed selective inhibitory activity for EGFR and VEGFR2 (IC_50_ = 6.17 and 0.09 μM, respectively) [27]. The mechanism responsible for the anti-VEGFR2 activity was assessed in that study by molecular docking simulation for compound 16 and sunitinib, to predict the protein–ligand interactions with the active VEGFR2 site. The findings showed that both compound 16 and sunitinib bind to the same active site on VEGFR2, suggesting that compound 16 could inhibit the same kinase target. Subsequently, compound 16 induced caspase-dependent apoptosis and reactive oxygen species (ROS) production in HeLa cells [27].

Given this background, compound 16 is of particular interest to the present study and is named HAA_2021_. The first objective of the present study is to investigate the cytotoxic activity and selectivity of HAA_2021_ against six cells, including MCF7 and MCF7/ADR cells and compare that activity with doxorubicin, followed by determination of the apoptotic activity of HAA_2021,_ doxorubicin and their combination in MCF7 and MCF7/ADR cells. The second objective is to investigate the P-gp and Hsp90 binding with HAA_2021_, and to determine whether the co-inhibition of P-gp and Hsp90 by HAA_2021_ contributes to the increase of the chemosensization of MCF7/ADR cells to doxorubicin, thus reversing the MDR of MCF7 cells and improving breast cancer chemotherapy.

## 2. Results

### 2.1. Synergistic Effects of Doxorubicin and HAA_2021_

#### 2.1.1. Cytotoxicity and Selectivity of Doxorubicin and HAA_2021_ against Six Cell-Lines

Determination of the cytotoxicity of doxorubicin against MCF7, HL60, K652, and HT29 cells showed IC_50_ in the range of 0.03–0.30 μM, with relative selectivity against cancer cells compared with the normal fibroblast MRC5 ranging from 1.66–16.66 (Table 1). However, the cytotoxicity of doxorubicin against MCF7/ADR cells decreased to 13.99 μM with selectivity index of only 0.03. The same table showed the cytotoxicity of HAA_2021_ against the four cancer cells (IC_50_: 0.22–16.04 μM, Table 1). MCF7 cells were the most sensitive to HAA_2021_ followed by HL60 cells. The relative selectivity of HAA_2021_ against cancer cells compared with MRC5 ranged from 1.19–86.59. Moreover, HAA_2021_ was less cytotoxic against MCF7/ADR cells compared with MCF7 cells and showed only 1.10 selectivity index, but MCF7/ADR cells were less resistant to HAA_2021_ compared with doxorubicin.

#### 2.1.2. Increased Chemosensitivity of MCF7/ADR Cells to Doxorubicin following Synergistic Combination with HAA_2021_

**Figure 2:**
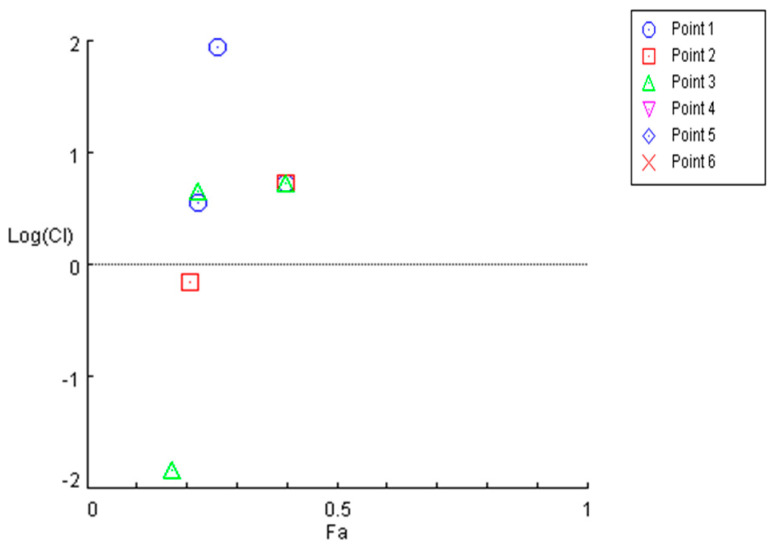
Non-constant drug ratio logarithmic combination index plot of doxorubicin and HAA_2021_ calculated using CompuSyn software. X-axis: Fraction affected (Fa: 0.5), y-axis: Log of combination index (CI). The six points in the legend corresponds with the CI values in Table 2. Green triangles: Combination of doxorubicin and 0.25 µM of HAA_2021_. Red rectangles: Combination of doxorubicin and 0.50 µM of HAA_2021_. Blue circles: Combination of doxorubicin and 1.00 µM of HAA_2021_. Plots are results of absorbance generated in three independent experiments (*n* = 3). CI > 1.1: Antagonism; CI = 0.9–1.1: Additive; CI = 0.8–0.9: Slight synergism; CI = 0.6–0.8: Moderate synergism; CI = 0.4–0.6: Synergism; CI = 0.2–0.4: Strong synergism. Plots were generated using CompuSyn software.

The cytotoxicity of HAA_2021_ in combination with different concentrations of doxorubicin was tested against MCF7/ADR cells. There were four points when the IC_50_ of the combination was less than the IC_50_ of doxorubicin or HAA_2021_ alone against MCF7/ADR cells. Consequently, the combination index (CI) of the respective four combinations was less than 1, showing a strong synergistic relationship between the two compounds (according to CompuSyn software version 1.0, CI = 0.8–0.9: Slight synergism; CI = 0.6–0.8: Moderate synergism; CI = 0.4–0.6: Synergism; CI = 0.2–0.4: Strong synergism). The least concentration showing synergism was doxorubicin (1.00 µM) and HAA_2021_ (0.25 µM) (Table 2, Figure 2). Therefore, HAA_2021_, at a non-toxic dose, increased the chemosensitivity of MCF7/ADR cells to doxorubicin.

#### 2.1.3. Synergistic Apoptotic Effect of Doxorubicin and HAA_2021_ in MCF7 and MCF7/ADR Cells

**Figure 3:**
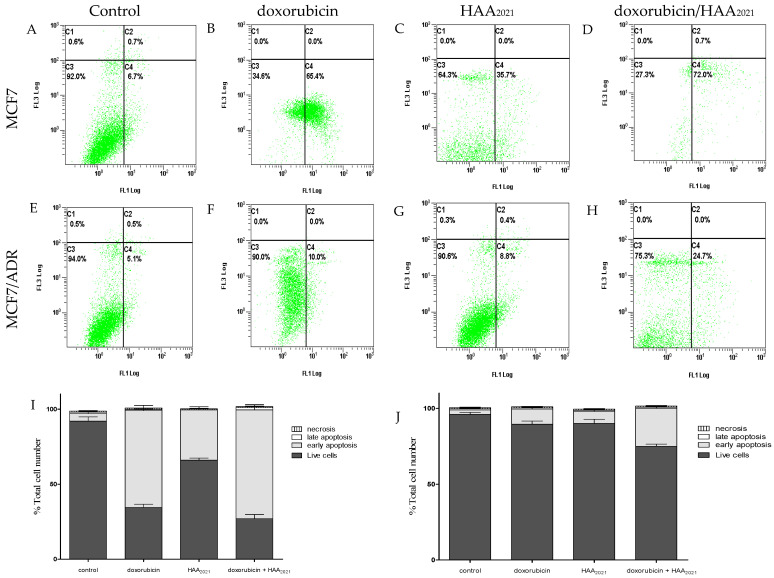
Induction of apoptosis in MCF7 and MCF7/ADR cells treated (72 h) with (**A**,**E**) vehicle control, (**B**,**F**) doxorubicin (1 µM), (**C**,**G**) HAA_2021_ (0.25 µM), and (**D**,**H**) doxorubicin/HAA_2021_ combination (1 and 0.25 µM, respectively). (**I**,**J**) Stacked histograms of the effect of doxorubicin and HAA_2021_ on MCF7 and MCF7/ADR cells. Results were expressed as mean ± SD, *n* = 3 × 3 independent experiments. C1: Necrosis, C2: Late apoptosis, C3: Live cells, C4: Early apoptosis.

The annexin V/FITC apoptosis assay was used to investigate the apoptotic effect of doxorubicin and HAA_2021_ and their combination in MCF7 and MCF7/ADR cells. The concentrations used were derived from Table 2, i.e., doxorubicin (1 µM) and HAA_2021_ (0.25 µM). The cells were exposed to the drug for 72 h, since at that time-point there is more possibility of drug resistance compared with the earlier time-points. Doxorubicin caused a significant induction of apoptosis in MCF7 cells, but it lost 6.5-fold of that activity against MCF7/ADR cells with the same dose/time of treatment (Figure 3B,F). Despite the fact that HAA_2021_ showed less effect on MCF7 and MCF7/ADR cells, respectively, it lost only 4.5-fold activity against MCF7/ADR cells compared with doxorubicin (Figure 3C,G). Interestingly, the combination of doxorubicin and HAA_2021_ caused more induction of apoptosis in MCF7 and MCF7/ADR cells compared with the effect of each compound alone, and the decrease of apoptosis in MCF7/ADR cells compared with MCF7 cells was down to less than 3-fold (Figure 3D,H). This result agrees with the previous cytotoxicity results, showing that HAA_2021_ increased the chemosensitivity and apoptosis inducing ability of MCF7/ADR cells to doxorubicin.

### 2.2. Inhibition of P-gp by HA_2021_

#### 2.2.1. Molecular Modeling of HA_2021_/P-gp

**Figure 4:**
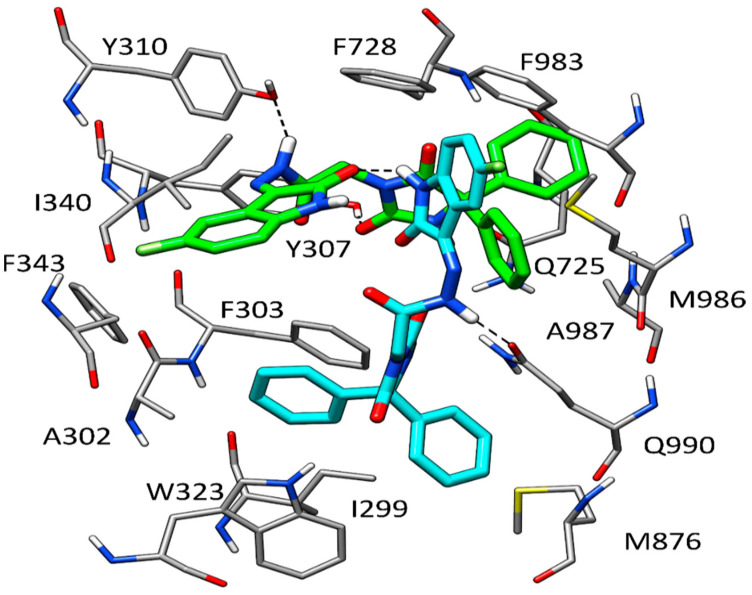
Binding mode predicted for two molecules of HAA_2021_ within P-gp.

In order to evaluate the potential binding mode of HAA_2021_ within P-glycoprotein (P-gp), molecular modeling studies were performed employing the robust docking procedure. The cryo-EM structure of human P-gp in complex with the known inhibitor zosuquidar was used for this study (PDB code 7A6F) [28]. In this case, the docking protocol identified a reliable cluster of solutions (see Section 4.2.4 for details). Therefore, the docking pose belonging to this cluster associated with the best estimated binding energy was selected as a representative binding mode. Recent studies demonstrated that, while a single molecule of P-gp substrates, such as vincristine, exclusively interacts with the central pocket of the protein, two different molecules of inhibitors, such as zosuquidar, elacridar, and tariquidar interact with the receptor at the same time, occupying also additional portions of the binding site, which would justify their inhibitory effect [28]. For this reason, we envisioned that two molecules of HAA_2021_ would bind to P-gp at the same time, as well. Therefore, the predicted HAA_2021_-P-gp complex was used for a second docking study aimed at predicting the binding disposition within the receptor of a second molecule of the ligand. Once again, a reliable cluster of solutions was identified, and the corresponding best energy pose was selected as the representative binding mode. As shown in Figure 4, the two molecules of HAA_2021_ are disposed perpendicularly to each other and interact both with the surrounding protein residues and with each other. A first ligand molecule (shown in green in Figure 4) occupies a wide portion of the central pocket of the protein, forming an h-bond with Y310 and a second one with Y307. The diphenylidantoin core of the molecule shows extensive hydrophobic interactions with the side chains of Q725, F728, F983, M986, and A987, while the isatin moiety interacts with I340 and F343. The second molecule of HAA_2021_ (shown in cyan in Figure 4) occupies the central pocket of the protein with the isatin-derived moiety, which forms lipophilic interactions with F983 and M986, as well as an h-bond with the carbonyl group of the isatin moiety of the first ligand molecule. Moreover, the central portion of the ligand forms an additional h-bond with the side chain of Q990. Finally, the diphenylidantoin core of the second inhibitor molecule extends into the vestibule of P-gp binding site, forming a double π-π stacking with F303 and W323, as well as hydrophobic interactions with I299, A302, M876, and Q990. 

#### 2.2.2. Concentration-Dependent Inhibition of P-gp in MCF7/ADR Cells by HAA_2021_ Using Rho123 Efflux and Accumulation Assays

**Figure 5:**
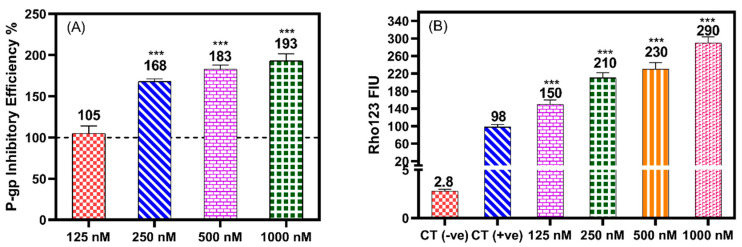
The effect of different concentrations HAA_2021_ on the P-gp transporter protein in MCF7/ADR cells. (**A**) The Rho123 efflux assay. Data expressed as mean ± SD of P-gp inhibitory efficiency compared with verapamil as a positive control (100%) as represented as the dashed line to show the decrease of efflux. (**B**) Accumulation assay. Rho123-FIU was used to compare the Rho123 fluorescence accumulation by HAA_2021_ with the untreated control (CT −ve), verapamil positive control (CT +ve) in MCF7/ADR, to show the increase of doxorubicin accumulation. *** *p* < 0.001 indicated the significant difference of treated cells compared with verapamil.

The rhodamine-123 (Rho123) efflux assay was performed to test the activity of HAA_2021_ against P-gp in MCF7/ADR cells. Rho123 is a fluorescent dye which is a known substrate for P-gp. The effect of several concentrations of HAA_2021_ was compared with the effect of verapamil as a positive control for P-gp inhibition. HAA_2021_ (125–1000 nM) showed significant inhibitory effects on the efflux of Rho123 from MCF7/ADR cells in a concentration-dependent manner, (1.05-, 1.68-, 1.83-, and 1.93-fold increase compared with verapamil, Figure 5A). To further confirm the HAA_2021_ P-gp-modulatory effect, the Rho123 accumulation assay was carried out with MCF7/ADR cells. After the efflux period, the Rho123 fluorescence was quantified by a spectrofluorometer. HAA_2021_ (125–1000 nM) significantly increased the intracellular fluorescence in a dose-dependent manner in MCF7/ADR cells (150–290 FIU: fluorescence intensity unit) more than verapamil (98 FIU) (Figure 5B).

### 2.3. Inhibition of Hsp90α by HA_2021_

#### 2.3.1. Surface Plasmon Resonance Analyses of HA_2021_/Hsp90α

The interaction between HA_2021_ with Hsp90α (full length protein) was investigated by a surface plasmon resonance-based (SPR) binding assay [29,30,31,32,33]. Radicicol and 17-*N*-allylamino-17-demethoxygeldanamycin (17-AAG) [34,35,36] were chosen as positive controls. It was found that HA_2021_ interacted efficiently with the immobilized protein. As a result of fitting the relative sensorgrams to a single-site bimolecular interaction model, the thermodynamic parameters for the resulting complex formation were determined.

**Figure 6:**
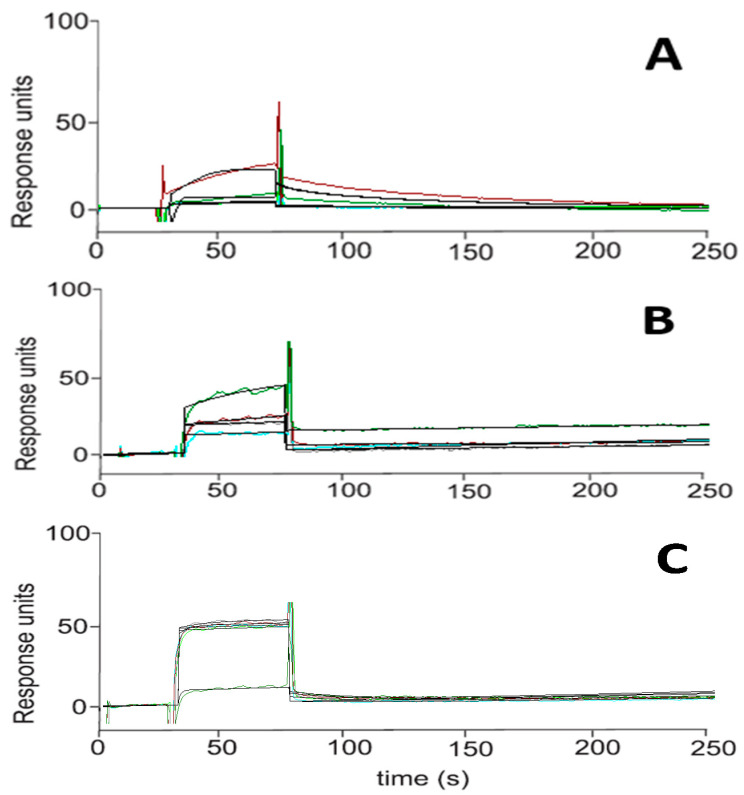
Surface plasmon resonance sensorgram acquired for HAA_2021_ (**A**) interacting with Hsp90α and for the positive controls radicicol (**B**) and 17-AAG (**C**). Each compound was injected onto the Hsp90α modified sensor chip at six different concentrations in the range of 0.025–4.000 μM. X-axis: time (s), y-axis: response units.

The analysis of the resulting sensorgram (Figure 6) clearly showed a significant reversible interaction of HAA_2021_ and 17-AAG with the protein, as demonstrated by the concentration-dependent responses (ranging from 0.025 to 4 μM), and by the clearly discernible exponential curves, during both the association and dissociation phases (Figure 6A,C). These data indicate that compounds HAA_2021_ and 17-AAG have a similar binding mode towards Hsp90α, whereas the radicicol sensorgram (Figure 6B) showed parallel curves in the dissociation phase, thus indicating that the complexes formed by the interaction of the protein were extremely stable. The results are consistent with the SPR results obtained with the full-length protein (Table 3). This approach allowed the measurement of 41.20 nM K_D_ (equilibrium dissociation constant) for the Hsp90α/HAA_2021_ complex compared with 1.20 nM K_D_ for the Hsp90α/radicicol complex. Interestingly, HAA_2021_ showed an affinity towards the chaperone that was greater than determined for 17-AAG (K_D_ = 360.00 nM) (Table 3, Figure 6).

#### 2.3.2. Molecular Modeling of HA_2021_/Hsp90α

With the aim of providing a possible model for the interaction between HAA_2021_ and Hsp90α, docking studies followed by molecular dynamic (MD) simulation and relative binding energy evaluations were performed. X-ray structures of Hsp90α show remarkable plasticity, particularly in residues 104–111 located in α-helix3, that can adopt “loop-in” or “loop-out” conformations. Furthermore, recent studies have revealed ligands occupying an additional binding subpocket created by the rearrangement of residues 104–111 into a continuous helical conformation [37]. On this basis, a representative structure of the “loop-in”, “loop-out”, and “helical” conformations was considered. As a first step of our analysis, HAA_2021_ was docked into the three protein conformations by applying a robust AutoDock procedure that had previously shown good results in virtual screening studies and in the prediction of ligand’s binding poses [38,39]. For each of the three protein conformations, the 200 different docking results generated were clustered using a root-mean-square deviation (RMSD) threshold of 2.0 Å. In total, 11 clusters of solutions were thus identified and considered for further studies: Five clusters for the “helix”, four for the “loop-in”, and two for the “loop-out” protein conformation (Section 4.2.4). For each cluster, the docking pose associated with the best estimated binding energy was selected as a representative binding mode.

**Figure 7:**
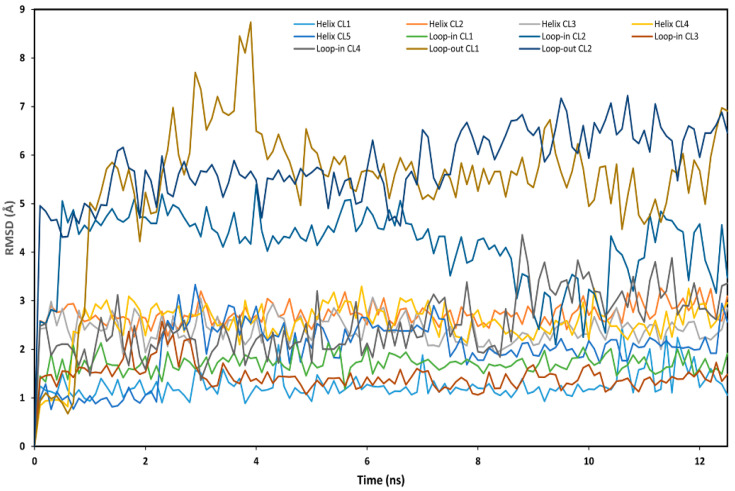
Analysis of the MD simulations of the eleven different Hsp90/HAA_2021_ complexes. The RMSD of the position of the ligand with respect to its initial docking pose is shown.

Then, the stability of the 11 different binding modes was assessed by means of MD simulations. The different complexes were subjected to a total of 12.5 ns of MD simulation and the RMSD of the ligand’s position with respect to the original docking pose was analyzed. Although in each complex the ligand showed at least some adjustment of its binding pose, in the case of cluster 2 of the “loop-in” and cluster 1 and 2 of the “loop-out” protein conformation, the ligand showed greater RMSD fluctuation (Figure 7), suggesting that the ligand was endowed with a higher freedom of movement inside the binding site, and thus maintained the binding disposition with a low stability. On the contrary, cluster 1 and 5 of the “Helix” and cluster 1 and 3 of the “loop-in” protein conformation showed the higher stability with and average RMSD value smaller than 2.0 Å.

To better assess the reliability of the different Hsp90/HAA_2021_ binding complexes, the corresponding ligand-protein interaction energies were evaluated from the MD coordinates extracted from the last 10 ns of simulation. The molecular mechanics-generalized born surface area (MM-GBSA) and the molecular mechanics-Poisson Boltzmann surface area (MM-PBSA) methods, reliably assessing the binding energy interaction, as shown in [40,41,42], were used for the calculation (see Section 4.2.4 for details).

These approaches analyze the MD simulation snapshots and calculate the contributions of both gas-phase and solvation free energies for unbound ligand, unbound protein, and bound complex. Then, the average contribution of each component is used to calculate the ligand-protein interaction energy. As shown in Table 4, the analysis identified cluster 1 of the “loop-in” protein conformation as the most reliable binding mode, since it showed the best binding energy according to both evaluation methods (ΔGBSA = −47.0 kcal/mol; ΔPBSA = −37.8 kcal/mol) and exceeded by at least 10 kcal/mol the interaction energies associated with the other poses.

**Figure 8:**
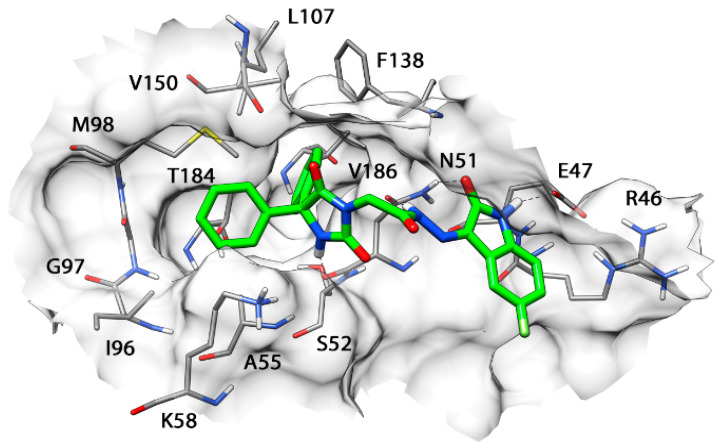
Minimized average structure of HAA_2021_ bound to Hsp90α.

Figure 8 illustrates the minimized average structure of HAA_2021_ complexed with Hsp90 (obtained from cluster 1 in the “loop-in” protein conformation) in the predicted binding mode obtained from the last 10 ns of MD simulation. The two phenyl rings are placed into a lipophilic cavity of the protein and show lipophilic interactions with I96, M98, L107, F138, V150, T184, and V186. The imidazolindione-2,4-dione ring shows an h-bond with the oxygen backbone of N51, whereas the 3-hydrazonioindolinone fragment shows two h-bonds with the sidechain of N51 and one h-bond with the sidechain of E47. An analysis of these four h-bonds suggests that these interactions are very stable as they were maintained for more the 85% of the whole MD simulation.

**Figure 9:**
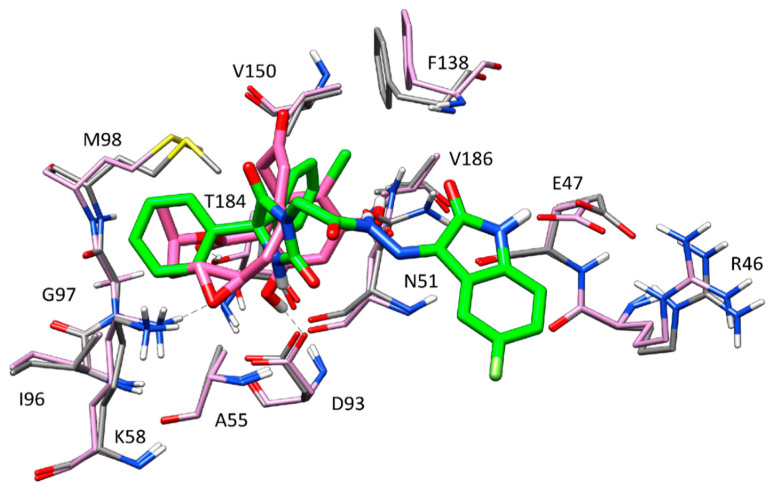
Superimposition of the HAA_2021_ binding mode with radicicol (PDB code: 4EGK) into Hsp90. The h-bonds between radicicol (dark pink) and the protein (light pink) are shown as black dashed lines.

**Figure 10:**
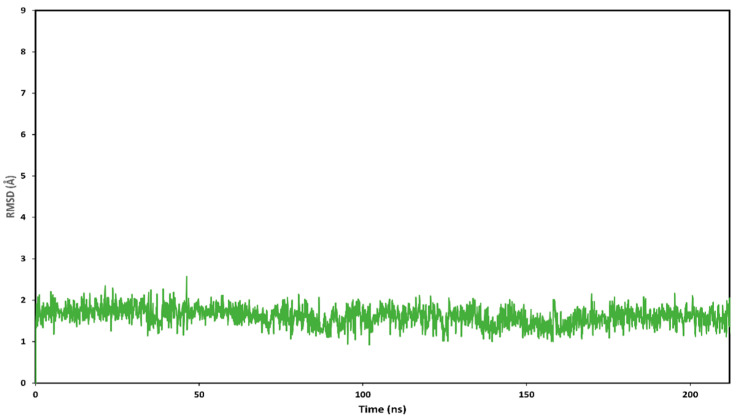
Analysis of the MD simulation of the predicted binding mode of HAA_2021_ complexed with Hsp90. The plot shows the RMSD of the ligand disposition during the simulation with respect to its initial docking pose.

**Figure 11:**
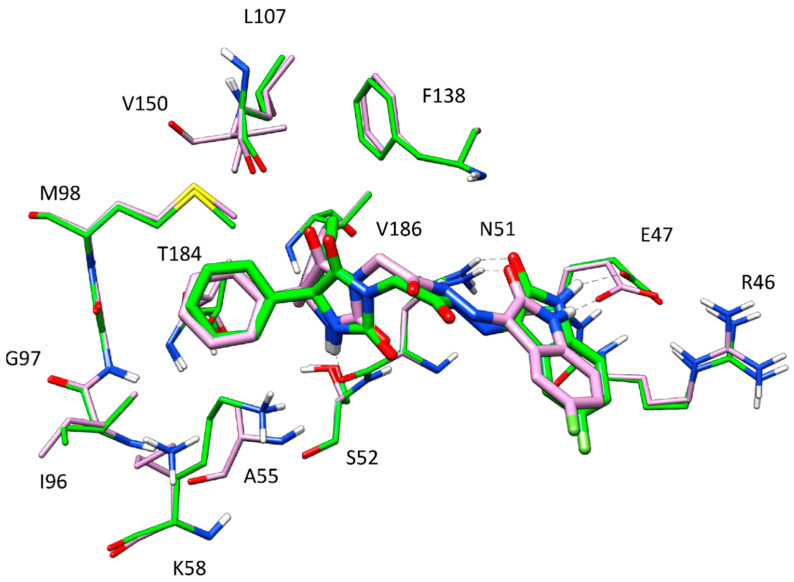
Superposition of the final frame of the elongated MD (shown in pink) with the predicted HAA_2021_-Hsp90 complex (shown in green).

By comparing the predicted binding mode of the ligand with the binding disposition of radicicol (PDB code: 4EGK), it can be seen that the two phenyl rings of HAA_2021_ are located within the same lipophilic cavity occupied by the cyclic core of Radicicol, which presents h-bonds with K58, T184, and D93 (Figure 9). Although HAA_2021_ does not form any h-bond within this cavity, it also interacts with an additional portion of the binding site and is well anchored to the protein thanks to the four h-bonds described above, thus justifying its high affinity towards the protein (K_D_ 41.20 nM), albeit reduced with respect to radicicol (K_D_ 1.80 nM). To further demonstrate the reliability of the predicted binding mode of HAA_2021_, the MD simulation of the corresponding Hsp90-HAA_2021_ complex was extended to 200 ns. The results confirmed the strong stability of both the binding disposition of the ligand, with an RMSD value below 2.0 Å (Figure 10), and its interactions with the protein (Figure 11).

#### 2.3.3. HAA_2021_ Inhibits Hsp90α and NF-κB in MCF7 and MCF7/ADR Cells

**Figure 12:**
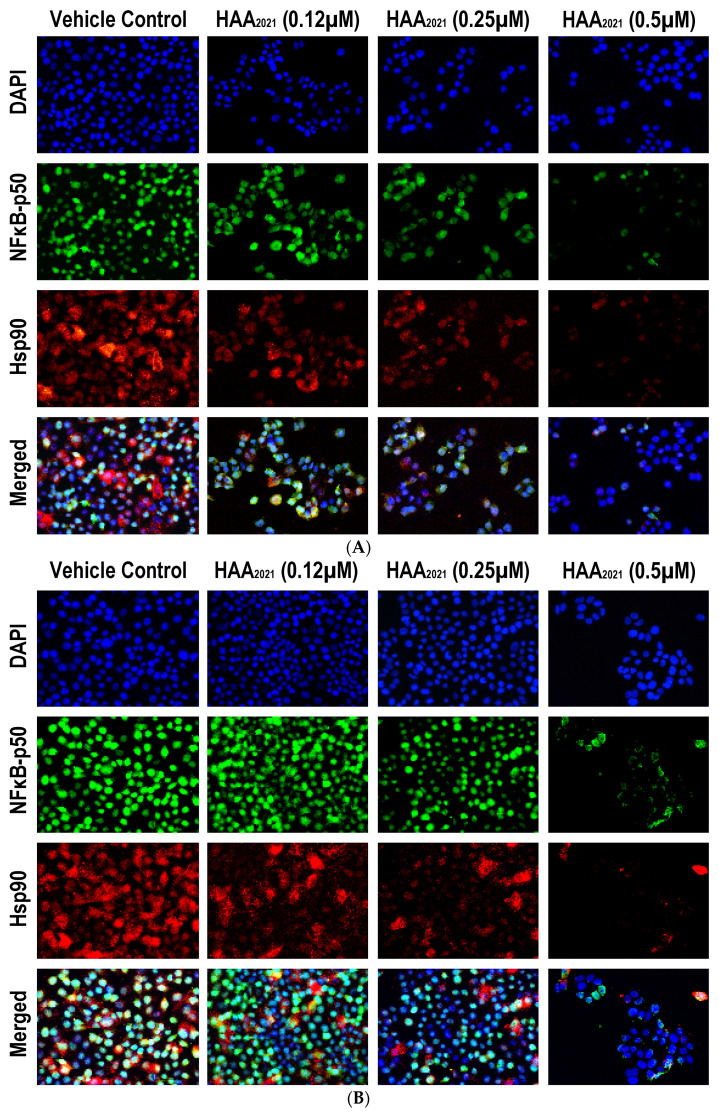

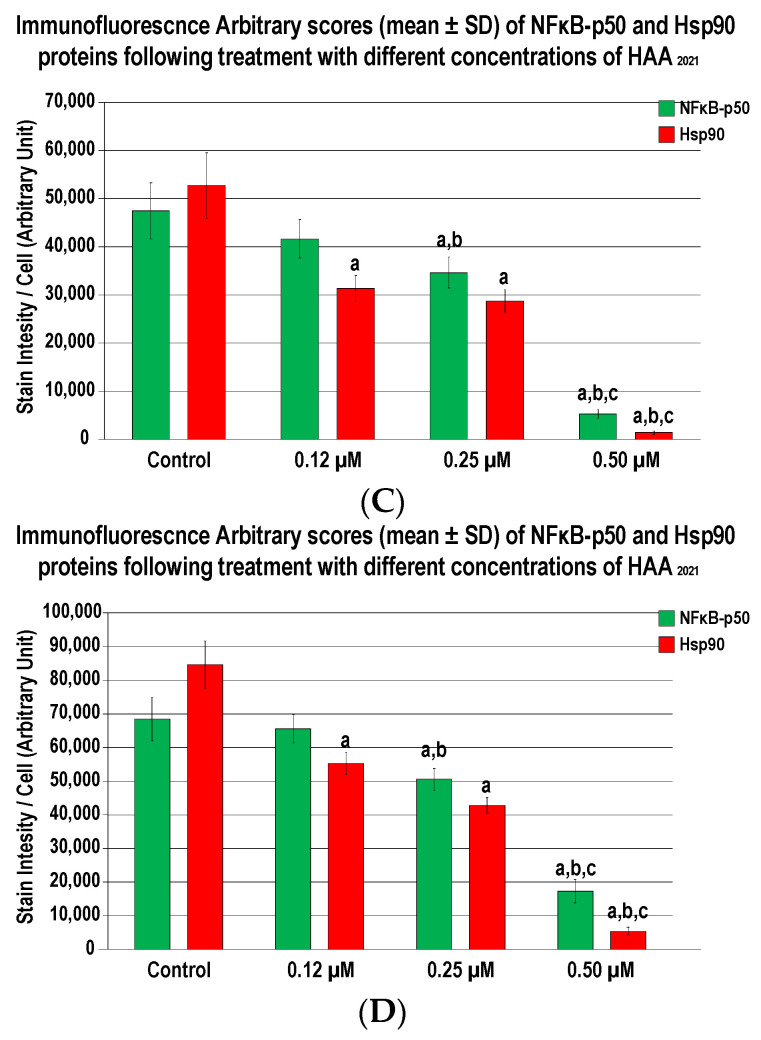
Double localization of NF-κB (green) with Hsp90α (red) by immunofluorescence in the MCF7 (**A**) and MCF7/ADR cells (**B**) following treatments with different concentrations of HAA_2021_ for 72 h (40× objective; scale bar = 10 µm). Arbitrary scores of the immunofluorescent stain intensity/MCF7 cells (**C**) and MCF7/ADR cells (**D**) (mean ± SD) for each protein are shown as graph bars (a = *p* < 0.05 compared with the control untreated cells; b = *p* < 0.05 compared with the 0.12 µM treatment; and c = *p* < 0.05 compared with the 0.25 µM treatment).

Using the immunofluorescence staining method, the protein expression of NF-κB (green) and Hsp90α (red) were detected in the untreated MCF7 and MCF7/ADR cells. Both proteins showed strong staining intensities (Figure 12). Cells treated with 0.12 µM of HAA_2021_ disclosed a marked decrease in the Hsp90α, but not the NF-κB, relative to the untreated cells. In contrast, the protein expression of NF-κB declined significantly with the 0.25 µM concentration compared with the non-treated cells and the cells treated with 0.12 µM of HAA_2021_, whereas the Hsp90α staining intensities were comparable between the 0.25 and 0.12 µM treatments. On the other hand, the lowest significant immunostaining intensities for both proteins were observed with the 0.50 µM compared with all groups. HAA_2021_ showed more inhibition of Hsp90α and NF-κB in MCF7 compared with the MCF7/ADR cells.

### 2.4. Synergistic Inhibition of P-gp/Hsp90α in MCF7/ADR Cells by Doxorubicin and HAA_2021_

**Figure 13:**
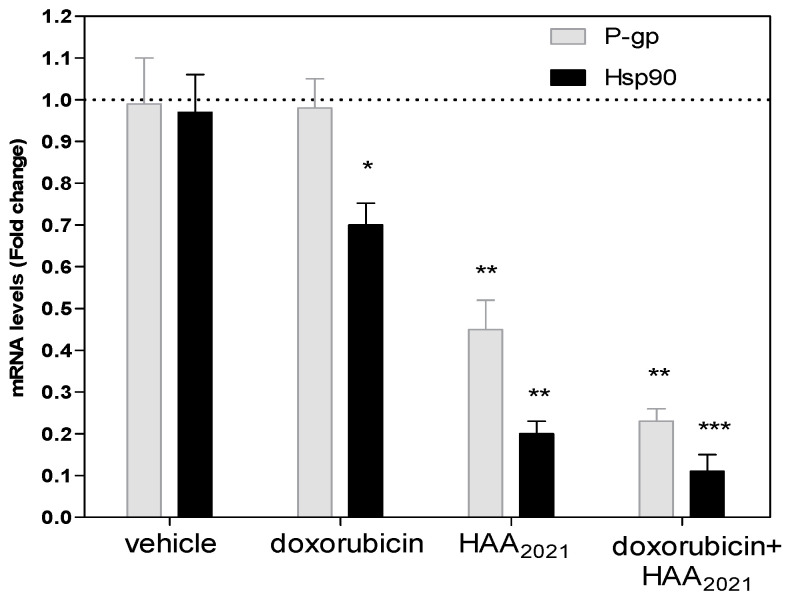
The inhibitory effect (72 h) of vehicle control, doxorubicin (1 µM), HAA_2021_ (0.25 µM) or their combination on the expression of mRNA of P-gp in MCF7/ADR cells was quantified by RT-PCR. The data represent the mean ± SD of the fold change related to the vehicle control (fold change = 1 dashed line, *n* = 2 × 2 independent experiments). *p* < 0.05 (*), *p* < 0.01 (**), and *p* < 0.001 (***) were considered significant.

Regarding the results of the present study, HAA_2021_ was proven to cause the inhibition of P-gp and Hsp90 in MCF7/ADR cells. To confirm the simultaneous effect of HAA_2021_ on these two proteins, the mRNA amount of P-gp and Hsp90 genes were assessed by real-time-PCR following the treatment of MCF7/ADR cells with the vehicle control, doxorubicin (1 µM), HAA_2021_ (0.25 µM), and their combination (doxorubicin 1 µM, HAA_2021_ 0.25 µM, respectively) for 72h. Doxorubicin alone caused no inhibition of P-gp, while HAA_2021_ when combined with doxorubicin caused a significant inhibition of P-gp (Figure 13). However, doxorubicin caused a significant inhibition of Hsp90 gene, and similarly HAA_2021_ and its combination with doxorubicin caused the highly significant inhibition of Hsp90. The combination of HAA_2021_ and doxorubicin caused a synergistic inhibition of P-gp and Hsp90 gene expression in MCF7/ADR cells.

## 3. Discussion

Naturally occurring isatins and their derived compounds, both as multi-target or combined-agents, have established anticancer activities supported by the approval of FDA for many anticancer drugs, including sunitinib, osimertinib, alictinib, and panobinostat [15].

The cytotoxicities of HAA_2021_ and doxorubicin were tested in the present study against six cells using the MTT assay. Then, HAA_2021_ and doxorubicin were combined to examine the possibility of chemosensitizing MCF7/ADR cells to doxorubicin, as the later IC_50_ against MCF7/ADR cells was only 13.99 μM compared with 0.05 μM against MCF7 cells. Therefore, doxorubicin lost around 280-fold of its activity to MCF7/ADR cells. Interestingly, HAA_2021_ has previously shown cytotoxicity against three cells, including MDA-MB-231 breast cancer cells [27]. In the present study, HAA_2021_ exhibited cytotoxicity against five cancer cells, with the most significant effect against MCF7 breast cancer cells and the least against MCF7/ADR breast cancer cells (IC_50_: 0.22 and 17.21 μM, respectively). However, the MCF7/ADR cell line was less resistant to HAA_2021_ compared with doxorubicin, as HAA_2021_ showed 1.10 selectivity compared with only 0.03 by doxorubicin for MCF7/ADR cells. The CI of HAA_2021_ and doxorubicin against MCF7/ADR cells showed a synergistic relationship (0.001–0.692) in four concentration points (according to CompuSyn software, CI = 0.8–0.9: Slight synergism; CI = 0.6–0.8: Moderate synergism; CI = 0.4–0.6: Synergism; CI = 0.2–0.4: Strong synergism). The least concentration showing synergism was doxorubicin/HAA_2021_: 1 µM /0.25 µM. Therefore, HAA_2021_, at non-toxic doses, increased the chemosensitivity of MCF7/ADR cells to doxorubicin. Additionally, in agreement with that result, the combination of doxorubicin/HAA_2021_ (1 µM /0.25 µM) also caused the synergistic induction of apoptosis in MCF7/ADR cells using the annexin V/FITC assay.

Next, we performed molecular modeling studies for prediction of the interaction mode between HAA_2021_ and P-gp, which showed that two molecules of HAA_2021_ would bind to P-gp at the same time, and are disposed perpendicularly to each other, thus interacting with the surrounding protein residues and with each other. To confirm this relationship with another cell-based assay, HAA_2021_ was found to cause a concentration-dependent P-gp inhibitory effect on the MDR efflux pump compared with verapamil. Consequently, HAA_2021_ increased the MCF7/ADR intracellular fluorescence and accumulation of Rho123 in a dose-dependent manner, as compared with verapamil again. Similarly, in a previous study, the synthesized N-alkylated isatins were found to decrease the P-gp mediated efflux in the MES-SA/Dx5 MDR cells, resulting in apoptosis [43].

In the next step, we performed studies to investigate the possible interaction between HAA_2021_ and Hsp90 [30,31,33,39]. First, the SPR technique was used, which showed that HAA_2021_ interacted efficiently with the immobilized Hsp90α protein at 41.20 nM K_D_, which was significantly better than the interaction between Hsp90 and 17-AAG (K_D_ = 360.00 nM). Second, we used a molecular docking model followed by molecular dynamic (MD) simulation to study the interaction mode between HAA_2021_/Hsp90. The study showed that the two phenyl rings of HAA_2021_ are placed into a lipophilic cavity of the protein and showed hydrophobic interactions with I96, M98, L107, F138, V150, T184, and V186. Moreover, the imidazolindione-2,4-dione ring showed an h-bond with the oxygen backbone of N51, whereas the 3-hydrazonioindolinone fragment showed two h-bonds with the sidechain of N51 and one h-bond with the sidechain of E47. This all suggest that the interactions are very stable as they were maintained for more than 85% of the whole MD simulation. As a conformational step for the Hsp90 inhibition activity, we used the cell-based immunofluorescence staining, which was employed to detect the protein expression of Hsp90 and NF-κB in MCF7 and MCF7/ADR cells following the treatment with HAA_2021_. The treated cells showed significant decreases in the Hsp90 and NF-κB proteins relative to the untreated cells, while proteins were more inhibited in MCF7 cells compared with MCF7/ADR cells. It was previously documented that the inhibition of Hsp90 causes the deactivation of NF-𝜅B through many mechanisms, including the disassociation of the inhibitor of I𝜅B kinase (IKK) [44]. Finally, while doxorubicin alone did not show an inhibiting effect on P-gp at 1 µM, we confirmed that the combination of doxorubicin/HAA_2021_ (1 µM/0.25 µM, respectively) synergistically co-inhibited the expression of P-gp/Hsp90α genes in MCF7/ADR cells using RT-PCR assays.

According to the present study, combining HAA_2021_, at a non-toxic dose, with doxorubicin synergistically inhibited the proliferation while inducing significant apoptosis in MCF7 cells. HAA_2021_ also increased the chemosensitization of MCF7/ADR cells to doxorubicin, paving the way for better cytotoxicity/ selectivity and apoptosis-inducing efficiency compared with the effect of each compound against MCF7/ADR cells. Exploring the molecular modeling apoptotic pathways showed that two molecules of HAA_2021_ would bind to P-gp at the same time, causing a concentration-dependent P-gp inhibitory effect on the MDR efflux pump and the accumulated Rho123 in MCF7/ADR cells.

HAA_2021_ stably interacted with Hsp90α more efficiently compared with 17-AAG, as confirmed with the SPR and molecular modeling studies. Additionally, HAA_2021_ showed multi-target effects via the inhibition of Hsp90 and NF-𝜅B proteins both in MCF7 and MCF7/ADR cells. In a study, the NF-𝜅B was found to be involved in the activation of MDR expression in cancer cells [45]. Moreover, the inhibition of Hsp90 was documented to contribute to the reversal of cancer MDR [46,47].

**Figure 14:**
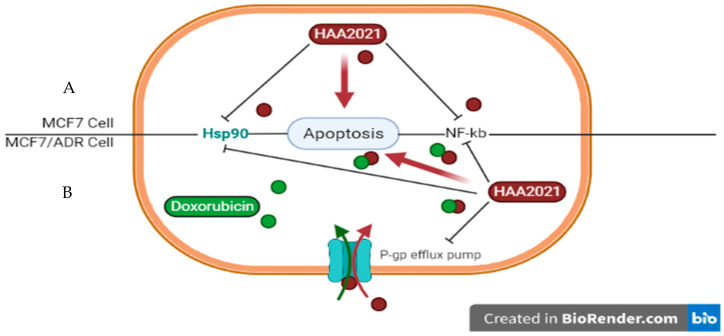
(**A**) HAA_2021_ inhibits the expression of Hsp90 and NF-kB in MCF7 cells leading to apoptosis. (**B**) The chemosensitizing effect of HAA_2021_ on MCF7/ADR cells to doxorubicin. The combination of HAA_2021_/doxorubicin enhances the doxorubicin-resistance, downregulates the P-gp protein synthesis and expression, and inhibits its efflux pump action, thus improving the accumulation of doxorubicin, and potentiating its targets on MCF7/ADR cells leading to apoptosis.

Many cancers can be considered in general as complex and multi-drug resistance (MDR) diseases, which necessitate continuous discovery and derivatization of effective molecules [12,13,14,16,17]. HAA_2021_, whether combined with doxorubicin or alone, has proven in the present study to have apoptotic, chemosensitizing/MDR reversal activity in MCF7/ADR cells, in addition to its Hsp90/ NF-𝜅B inhibitory activities in MCF7 cells (Figure 14). The molecular docking and molecular dynamic simulation studies performed were supported by in vitro data in five cancer cells in the present study, including doxorubicin-sensitive (MCF7) and -resistant (MCF7/ADR) cell lines. Many Hsp90 inhibitors are expelled off their target cells by members of the ABC transporters, especially P-gp [48]. Proving that HAA_2021_, as an inhibitor of Hsp90, is not affected by the pumping out of the cancer cell by the P-gp could be an interesting point for the development of this compound. Further pharmacokinetic and in vivo studies for HAA_2021_ are warranted to guarantee its anticancer capabilities.

## 4. Materials and Methods

### 4.1. Chemicals and Reagents

All of the chemicals and reagents were obtained from Merk and Sigma-Aldrich Co. (St. Louis, MO, USA), unless another supplier is mentioned in the manuscript. HAA_2021_ was kindly provided by collaborators from King Saud University, Saudi Arabia (Figure 1, [27]).

### 4.2. Methods

#### 4.2.1. Cells and Maintenance

Five cancer cell lines: MCF7, MCF7/ADR, HT29, HL60, and K562 were used in the present study, in addition to the MRC5 normal fibroblast. All of the cells, except MCF7/ADR, were obtained from the ATCC (Manassas, VA, USA). The five cancer cells were maintained in RPMI-1640 media (10% FBS), while the MRC5 was maintained in Eagles minimum essential medium (EMEM, 10% FBS), with all of the cells containing 1% antibiotic-antimycotic. CO_2_ incubator conditions: 37 °C, 5% CO_2_, and 100% relative humidity [49]. MCF7/ADR cell line was sub-cultured in RPMI-1640 media containing gradually added doxorubicin (up to 5 µg/mL) under the same above conditions. Doxorubicin was excluded from subcultures 5 days prior to the experiments.

#### 4.2.2. MTT Cytotoxicity, Selectivity, and Combination Assays

Following the previously reported methods [50,51], the cytotoxicity of HAA_2021_ was evaluated by the MTT assay. The five cell lines and one normal fibroblast cell were separately cultured in 96-well plates (3 × 10^3^/ well), and incubated at 37 °C overnight. Final HAA_2021_ concentrations: 0.00–50.00 μM in triplicates. The plates were incubated for 72 h, followed by the addition of MTT to each well. Next, the plates were incubated for 3 h, the supernatant was aspirated, the DMSO was added to each well, and the absorbance was read on a multi-plate reader. The HAA_2021_ concentration causing 50% inhibition (IC_50_) compared with the control cell growth (100%) was determined. The selectivity index (SI) was calculated by dividing the IC_50_ of MRC-5 cells by the IC_50_ of either of the used cells. In the combination study, the MCF7/ADR cells were treated with different concentrations of doxorubicin and HAA_2021_ for 72 h. Next, the IC_50_ value of each point was used for the calculation of the combination index (CI) using the CompuSyn software, as previously described [52].

#### 4.2.3. Determination of Apoptosis by Flowcytometery

The annexin V/FITC double staining assay was used to test the possible apoptosis inducibility of MCF7 and MCF7/ADR cells (1 × 10^6^ cells/ 6-well plate) treated with the vehicle control (0.00 µM), doxorubicin (1 µM), HAA_2021_ (0.25 µM) or their combination for 72 h. Annexin V and propidium iodide (Invitrogen, Waltham, MA, USA) were used according to a previous report [53]. The samples were finally investigated by flowcytometery using the Beckman coulter flow cytometer (BC, FC500).

#### 4.2.4. Molecular Docking Calculations

The crystal structure of the “loop-in”, “loop-out”, and “helical” conformations of Hsp90 protein (5J64, 1YET, and 2WI7 PDB codes, respectively) were taken from the Protein Data Bank [54]. After adding hydrogen atoms, the proteins were minimized using the Amber16 software and the ff14SB force field at 300 K. The complexes were placed in a rectangular parallelepiped water-box, an explicit solvent model for water, TIP3P, was used and the complex was solvated with a 20 Å water cap. Sodium ions were added as counter ions to neutralize the system. Then, two steps of minimization were carried out. In the first stage, we kept the protein fixed with a position restraint of 500 kcal/molÅ^2^ and we solely minimized the positions of the water molecules. In the second stage, we minimized the entire system through 5000 steps of the steepest descent followed by the conjugate gradient (CG) until a convergence of 0.05 kcal/Å·mol. The ligand was built using Maestro and was minimized by means of Macromodel in a water environment using the CG method until a convergence value of 0.05 kcal/Å·mol, using the MMFFs force field and a distance-dependent dielectric constant of 1.0. The AutoDock 4.0 software [55] was employed for molecular docking. The identification of the torsion angles in the ligands, the addition of the solvent model, and the determination of protein and ligand atomic charges was carried out using AutoDock tools. Kollmann charges were assigned to the protein and Gasteiger charges to the ligand. A grid spacing of 0.375 Å and a distance-dependent function of the dielectric constant were used for the energetic map calculations. The compounds were subjected to a robust docking procedure by applying 200 runs of AutoDock search, using the Lamarckian Genetic Algorithm with 10,000,000 steps of energy evaluations [56]. The number of individuals in the initial population was set to 500 and a maximum of 10,000,000 generations were simulated during each docking run. Cluster analysis was performed on the results using an RMS tolerance of 2.0 Å. Only the binding modes populated for more than 10% in the corresponding clusters of poses were considered, for a total of 11 different clusters.

The docking studies aimed at evaluating the potential binding mode of HAA_2021_ within P-glycoprotein, were performed using the cryo-EM structure of human P-gp in complex with the known inhibitor zosuquidar (PDB code 7A6F) [28] employing the same robust docking procedure used for the docking studies within Hsp90α. In this case, one of the generated clusters of solutions showed the highest population and the best binding energy. Therefore, it was selected as the most reliable cluster of solution. The docking of the second molecule of HAA_2021_ was performed in the P-gp-HAA_2021_ complex predicted through the first docking study. The cluster of solution showing the highest population and the best binding energy was again selected as the most reliable one.

##### Molecular Dynamic (MD) Simulations

All of the simulations were performed using AMBER, version 16 and were carried out using the ff14SB force field at 300 K. General Amber force field (GAFF) parameters were assigned to the ligand, while partial charges were calculated using the AM1-BCC method with the Antechamber suite of AMBER 16. Using the TIP3P explicit solvent model, a 20 Å water cap was generated around the complexes, which was thus placed at the center of a rectangular parallelepiped box of explicit water molecules. Then, sodium ions were added for the neutralization of the systems. Prior to MD simulations, two steps of energy minimization were performed with the same procedure described above. The minimized structures of the complexes were used as the starting conformations for the MD simulations, which were run using Particle Mesh Ewald (PME) electrostatics and periodic boundary conditions. The time step of the simulations was 2.0 fs with a cutoff of 10 Å for the non-bonded interaction, while SHAKE was employed to keep all of the bonds involving the hydrogen atoms rigid. An initial MD step of 0.5 ns with constant-volume periodic boundary conditions was performed and the temperature of the system was raised from 0 to 300 K. Subsequently, a second step of constant pressure periodic boundary MD was run for 12 ns, keeping the temperature of the system at the constant value of 300 K with a Langevin thermostat. A harmonic potential of 10 kcal/mol·Å^2^ was applied on all α carbons of the protein during both MD steps. The final structure of HAA_2021_-Hsp90 complex corresponded to the average of the last 10.0 ns of MD minimized by the CG method until a convergence of 0.05 kcal/mol·Å^2^. The average structure was obtained using the CPPTRAJ program [57] implemented in AMBER 16.

##### Binding Energy Evaluation

The evaluation of the binding energy associated with the different ligand-protein complexes analyzed through MD simulations was carried out using AMBER 16, as already reported [58]. The trajectories relative to the last 10 ns of each simulation were extracted and used for the calculation, for a total of 100 snapshots (at time intervals of 100 ps). Van der Waals, electrostatic, and internal interactions were calculated with the SANDER module of AMBER 16, whereas polar energies were calculated using both the Generalized Born and the Poisson−Boltzman methods with the MM-PBSA module of AMBER 16. Dielectric constants of 1 and 80 were used to represent the gas and water phases, respectively, while the MOLSURF program was employed to estimate the nonpolar energies. The entropic term was considered as approximately constant in the comparison of the ligand−protein energetic interactions.

#### 4.2.5. Rhodamine123 Efflux Assay by Flowcytometery

Individual cell fluorescence measurements were carried out as previously described [59]. The efflux was induced by incubating MCF-7/ADR cells with a fluorescent marker at the intensity of 1 × 10^4^ cells. This intensity was utilized to compare different settings. Verapamil was used as a positive control since it was shown to greatly limit the active efflux of fluorescent substrate indicators mediated by P-gp. To quantify and compare the effects of various concentrations (125–1000 nM), the fluorescence intensity of treated cells was normalized by calculating the relative fluorescence intensity (inhibitory efficiency) as a percentage of the positive (verapamil = 100) and negative untreated controls. The investigation was carried out using the Beckman coulter flow cytometer (BC, FC500).

#### 4.2.6. Rhodamine123 Accumulation Assay by Spectrofluorometer

The settings for cell seeding and growth were identical to those used in the previously reported accumulation experiments [60]. MCF-7/ADR cells in 96-well plates were washed twice with PBS and then treated for 60 min at 37 °C with 1 g/mL Rho123 to load the cells. Following x2 washes with PBS, cells were incubated in media containing several concentrations of HAA_2021_ (125–1000 nM). After 120-min of incubation at 37 °C, cells were washed twice with a cold PBS. SpectraMaxII spectrofluorometer was used to detect the concentration-dependent effect of HAA_2021_ by measuring the fluorescence of Rho123 in cells. Individual cells’ Rho123 fluorescence intensity units were measured and compared with controls.

#### 4.2.7. Surface Plasmon Resonance Analyses

To investigate the interaction between HA_2021_ and Hsp90α, the surface plasmon resonance (SPR) analyses was performed using a Biacore 3000 optical biosensor equipped with research-grade CM5 sensor chips (GE Healthcare, Chicago, IL, USA), according to a previously detailed method [29]. Recombinant Hsp90 surfaces, a BSA surface, and an unmodified reference surface, were prepared. Proteins (100 μg/mL in 10 mM sodium acetate, pH 5.0) were immobilized on individual sensor chip surfaces at a flow rate of 5 μL/min to produce densities of 8−12 kRU. Compound HAA_2021_, 17-*N*-allylamino-17-demethoxygeldanamycin (17-AAG), and radicicol (two positive controls), were dissolved in DMSO. The six-point concentration series (0.025−4.000 μM) were prepared. Bioevaluation software 3.2. (GE Healthcare) was used to elaborate simple interactions, which were adequately fit to a single-site bimolecular interaction model (A + B = AB), yielding a single K_D_ sensorgram.

#### 4.2.8. Immunofluorescence Staining

Immunofluorescence staining was performed according to the previous methods [61,62]. Each of MCF7 or MCF7/ADR cells (2 × 10^3^/chamber) in the 8-well chamber slides were treated for 72 h with the different concentrations of HAA_2021_ (0.00–0.50 µM) followed by washing and fixation for 15 min with 4% paraformaldehyde (Santa-Cruz Biotechnology Inc., Dallas, TX, USA). Following a second wash, the cells were permeabilized with 0.25% Triton X100 for 20 min, washed twice with PBS, followed by blocking with a normal donkey serum for 30 min (Santa-Cruz Biotechnology Inc.). The dual protein expression of nuclear factor kappa B (NF-𝜅B) and heat shock protein-90α (Hsp90α) was performed in duplicate wells. All of the primary antibodies were from Thermo Fisher Scientific (San Jose, CA, USA). The primary mouse monoclonal IgG antibodies were used for the detection of NF-𝜅B (#MA5-15870), whilst Hsp90-α was detected by rabbit monoclonal IgG antibodies (#sc-8396). Both primary antibodies were concurrently added to two wells/slide (1:150 concentration for both) followed by 3 h of incubation at room temperature. The wells were washed, and the cells were then incubated for 60 min with a mixture of tagged highly cross-adsorbed secondary donkey anti-rabbit (#A-31572; Alexa Fluor 555) and anti-mouse (#A-21202; Alexa Fluor 488) IgG antibodies (Thermo Fisher Scientific: San Jose, CA, USA). The cells were counterstained with 4′,6-diamidino-2-phenylindole (DAPI; #D3571; Thermo Fisher Scientific). Then, the detachable plastic wells were removed, and the slides were cover-slipped with a permanent fluorescence mounting medium (#S3023; Dako, Santa Clara, CA, USA). All of the wells were observed with a Leica DMi8 microscope and digital images were captured within the same session from 10 random non-overlapping fields/well using a 40× objective. The IF staining intensities of each targeted protein were measured by the digital image analysis using the Image J software version 1.8.0. and are expressed as arbitrary units/cell numbers in analyzed images.

#### 4.2.9. Quantitative Real Time-PCR

RT-PCR (Applied Biosystems 7500 Fast Real Time PCR System, Waltham, MA, USA) was applied to quantify the gene expression of P-gp and Hsp90 in MCF7/ADR cells [63]. Briefly, MCF7/ADR cells (2 × 10^6^ cells/well) were cultivated in 6-well plates for 72 h, then cells were treated with vehicle control (0.00 µM), doxorubicin (1 µM), HAA_2021_ (0.25 µM) or their combination. Total RNA was isolated according to the manufacturer’s instructions. The RT-PCR experiment was conducted with a mixture of cDNA, 2X SYBR Green I Master mix, PCR-grade water, forward and reversed human primers of selective genes, and GAPDH as housekeeping gene (Applied-Biosystems, Thermo Fisher Scientific, Waltham, MA, USA) (Table 5).

#### 4.2.10. Statistics and Drawing

Graphpad Prism was used to assess the multiple comparison tests. The one-way analysis of variance (ANOVA) with Tukey’s post-hoc were used for the assessment of statistical differences. Figure 14 was made in Biorender.com.

## Figures and Tables

**Table 1:** Cytotoxic activity (MTT 72 h, IC_50_ ± sd μM) of doxorubicin and HAA_2021_ against five cell lines and one normal fibroblast.

Cell Line	Doxorubicin		HAA_2021_	
	IC_50_	SI ^a^	IC_50_	SI ^b^
MCF7	0.05 ± 0.00	10.00	0.22 ± 0.05	86.59
MCF7/ADR	13.99 ± 2.10	0.03	17.21 ± 2.33	1.10
HL60	0.30 ± 0.04	1.66	2.36 ± 0.69	8.07
K562	0.03 ± 0.00	16.66	16.04 ± 4.45	1.19
HT29	0.04 ± 0.00	12.50	5.06 ± 1.61	3.76
MRC5	0.50 ± 0.09	-	19.05 ± 1.03	-

SI: Selectivity index = IC_50_ value of doxorubicin ^a^ or HAA_2021_ ^b^ against normal MRC5 cells/ IC_50_ value of doxorubicin or HAA_2021_ against either of the other cells. (-): Not applicable.

**Table 2:** IC_50_ (µM) and combination index of MCF7/ADR cells treated (72 h) with different concentrations of doxorubicin and HAA_2021_.

Drug (µM)		IC_50_	CI ^a^	R ^b^
Doxorubicin	HAA_2021_			
-	0–50	17.21 ± 2.33	-	-
0–50	-	13.99 ± 2.10	-	-
0.25	0.25	15.09 ± 1.03	>100	0.97
0.50	0.25	14.34 ± 3.00	4.555	0.91
1.00	0.25	6.04 ± 1.01	0.014	0.97
0.25	0.50	14.64 ± 2.60	>100	0.89
0.50	0.50	5.54 ± 0.87	0.692	0.93
1.00	0.50	1.01 ± 0.12	0.003	0.90
0.25	1.00	14.09 ± 2.87	90.244	0.95
0.50	1.00	13.02 ± 1.99	3.670	0.93
1.00	1.00	0.89 ± 0.07	0.001	0.94

^a^ CI: Combination index (Fa = 0.5); ^b^, r: The linear correlation coefficient of the ME-plot, which signifies the conformity of the data with the mass-action law (an indication of how good the data are).

**Table 3:** Thermodynamic constants (mean ± sd) measured by SPR for the interaction between the tested compounds and immobilized Hsp90α.

Compound	K_D_ (nM) ^a^
HAA_2021_	41.20 ± 2.10
Radicicol	1.80 ± 0.40
17-AAG	360.00 ± 21.90

^a^ Results were given as the mean ± standard deviation.

**Table 4:** MM-GBSA and MM-PBSA results for the eleven different Hsp90-HAA_2021_ complexes. ΔGBSA and ΔPBSA are the sum of the van der Waals (VDW), electrostatic (ELE), as well as polar (EGB/EPB) and non-polar (ESURF/ENPOLAR) solvation free energy. Data are expressed as kcal·mol^−1^.

	**MM−GBSA Method**				
**Hsp90−HAA_2021_ Complex**	**VDW**	**ELE**	**EGB**	**ESURF**	**ΔGBSA**
**Hel. CL1**	−51.9	−8.3	33.8	−6.7	−33.1
**Hel. CL2**	−47.2	−33.0	49.3	−6.0	−36.9
**Hel. CL3**	−45.4	−26.1	44.6	−6.2	−33.2
**Hel. CL4**	−50.3	−8.8	34.1	−6.2	−31.1
**Hel. CL5**	−45.4	−10.1	33.3	−5.8	−28.1
**L.−in CL1**	−51.4	−40.7	51.3	−6.2	−47.0
**L.−in CL2**	−40.1	−41.2	56.3	−5.5	−30.4
**L.−in CL3**	−45.4	−47.9	69.0	−6.2	−30.5
**L.−in CL4**	−40.5	−18.2	35.7	−5.2	−28.1
**L.−out CL1**	−34.6	−18.7	36.2	−4.4	−21.6
**L.−out CL2**	−41.1	−1.6	23.9	−5.2	−23.9
	**MM−PBSA method**				
**Hsp90−HAA_2021_ Complex**	**VDW**	**ELE**	**EPB**	**ENPOLAR**	**ΔPBSA**
**Hel. CL1**	−51.9	−8.3	43.1	−5.1	−22.2
**Hel. CL2**	−47.2	−33.0	59.0	−4.8	−26.0
**Hel. CL3**	−45.4	−26.1	57.0	−4.9	−19.5
**Hel. CL4**	−50.3	−8.8	43.3	−4.8	−20.5
**Hel. CL5**	−45.4	−10.1	37.2	−4.8	−23.1
**L.−in CL1**	−51.4	−40.7	58.9	−4.6	−37.8
**L.−in CL2**	−40.1	−41.2	69.6	−4.4	−16.1
**L.−in CL3**	−45.4	−47.9	76.7	−4.4	−21.1
**L.−in CL4**	−40.5	−18.2	45.7	−4.0	−17.0
**L.−out CL1**	−34.6	−18.7	43.8	−3.9	−13.5
**L.−out CL2**	−41.1	−1.6	31.6	−4.1	−15.2

**Table 5:** Sequence of GAPDH, P-gp, and Hsp90α primers.

Gene	Sequence
GAPDH	F: AGGTCGGTGTGAACGGATTTG R: TGTAGACCATGTAGTTGAGGTCA
P-gp	F: TGCTCAGACAGGATGTGAGTTG R: AATTACAGCAAGCCTGGAACC
Hsp90α	F: TTGGTTACTTCCCCGTGCTG R: GCCTTTTGCCGTAGGGTTTC

## Data Availability

Data is contained within the article.

## Sample Availability

Samples of the compounds are not available from the authors.

## Abbreviations

17-AAG	17-N-allylamino-17-demethoxygeldanamycin
ABC	ATP-binding cassette transporters
CI	combination index
EGF	epidermal growth factors
EGFR	epidermal growth factor receptors
Hsp90	heat shock protein 90
NF-𝜅B	nuclear factor kappa B
PI3K	phosphoinositide-3-kinase
P-gp	P-glycoprotein
Rho123	Rhodamine-123
VEGF	vascular endothelial growth factors
VEGFR	vascular endothelial growth factor receptors

## References

[1] Talevi A. (2015). Multi-target pharmacology: Possibilities and limitations of the “skeleton key approach” from a medicinal chemist perspective. Front Pharm..

[2] Neophytou C.M., Trougakos I.P., Erin N., Papageorgis P. (2021). Apoptosis Deregulation and the Development of Cancer Multi-Drug Resistance. Cancers.

[3] Housman G., Byler S., Heerboth S., Lapinska K., Longacre M., Snyder N., Sarkar S. (2014). Drug resistance in cancer: An overview. Cancers.

[4] Yang M., Li H., Li Y., Ruan Y., Quan C. (2018). Identification of genes and pathways associated with MDR in MCF-7/MDR breast cancer cells by RNA-seq analysis. Mol. Med. Rep..

[5] Abdalla A.N., Malki W.H., Qattan A., Shahid I., Hossain M.A., Ahmed M. (2021). Chemosensitization of HT29 and HT29-5FU Cell Lines by a Combination of a Multi-Tyrosine Kinase Inhibitor and 5FU Downregulates ABCC1 and Inhibits PIK3CA in Light of Their Importance in Saudi Colorectal Cancer. Molecules.

[6] El-Azab A.S., Al-Dhfyan A., Abdel-Aziz A.A.M., Abou-Zeid L.A., Alkahtani H.M., Al-Obaid A.M., Al-Gendy M.A. (2017). Synthesis, anticancer and apoptosis-inducing activities of quinazoline–isatin conjugates: Epidermal growth factor receptor-tyrosine kinase assay and molecular docking studies. J. Enzym. Inhib. Med. Chem..

[7] Korashy H.M., Maayah Z.H., Al Anazi F.E., Alsaad A.M., Alanazi I.O., Belali O.M., Al-Atawi F.O., Alshamsan A. (2017). Sunitinib inhibits breast cancer cell proliferation by inducing apoptosis, cell-cycle arrest and DNA repair while inhibiting NF-κB signaling pathways. Anticancer Res..

[8] Xia L., Tan S., Zhou Y., Lin J., Wang H., Oyang L., Tian Y., Liu L., Su M., Wang H. (2018). Role of the NFκB-signaling pathway in cancer. OncoTargets Ther..

[9] Tsou S.H., Chen T.M., Hsiao H.T., Chen Y.H. (2015). A critical dose of doxorubicin is required to alter the gene expression profiles in MCF-7 cells acquiring multidrug resistance. PLoS ONE.

[10] Bruns A.F., Yuldasheva N., Latham A.M., Bao L., Pellet-Many C., Frankel P., Stephen S.L., Howell G.J., Wheatcroft S.B., Kearney M.T. (2012). A heat-shock protein axis regulates VEGFR2 proteolysis, blood vessel development and repair. PLoS ONE.

[11] Pandeya S.N., Smitha S., Jyoti M., Sridhar S.K. (2005). Biological activities of isatin and its derivatives. Acta Pharm..

[12] Fouche G., Cragg G.M., Pillay P., Kolesnikova N., Maharaj V.J., Senabe J. (2008). In vitro anticancer screening of South African plants. J. Ethnopharmacol..

[13] Ren X., Xie X., Chen B., Liu L., Jiang C., Qian Q. (2021). Marine Natural Products: A Potential Source of Anti-hepatocellular Carcinoma Drugs. J. Med. Chem..

[14] Ringel I., Horwitz S.B. (1991). Studies with RP 56976 (taxotere): A semisynthetic analogue of taxol. J. Natl. Cancer Inst..

[15] Chadha N., Silakari O. (2017). Indoles as therapeutics of interest in medicinal chemistry: Bird’s eye view. Eur. J. Med. Chem..

[16] Havrylyuk D., Kovach N., Zimenkovsky B., Vasylenko O., Lesyk R. (2011). Synthesis and anticancer activity of isatin-based pyrazolines and thiazolidines conjugates. Arch. Pharm..

[17] Singh H., Singh J.V., Gupta M.K., Saxena A.K., Sharma S., Nepali K., Bedi P.M.S. (2017). Triazole tethered isatin-coumarin based molecular hybrids as novel antitubulin agents: Design, synthesis, biological investigation and docking studies. Bioorg. Med. Chem. Lett..

[18] Hamed A.R., Abdel-Azim N.S., Shams K.A., Hammouda F.M. (2019). Targeting multidrug resistance in cancer by natural chemosensitizers. Bull. Natl. Res. Cent..

[19] Zhang L., Chen F., Wang J., Chen Y., Zhang Z., Lin Y., Zhu X. (2015). Novel isatin derivatives of podophyllotoxin: Synthesis and cytotoxic evaluation against human leukaemia cancer cells as potent anti-MDR agents. RSC Adv..

[20] Rajesh Kumar M., Violet Dhayabaran V., Sudhapriya N., Manikandan A., Gideon D.A., Annapoorani S. (2020). p-TSA. H_2_O mediated one-pot, multi-component synthesis of isatin derived imidazoles as dual-purpose drugs against inflammation and cancer. Bioorg. Chem..

[21] Havrylyuk D., Zimenkovsky B., Vasylenko O., Gzella A., Lesyk R. (2012). Synthesis of new 4-thiazolidinone-, pyrazoline-, and isatin-based conjugates with promising antitumor activity. J. Med. Chem..

[22] Medvedev A., Buneeva O., Gnedenko O., Ershov P., Ivanov A. (2018). Isatin, an endogenous nonpeptide biofactor: A review of its molecular targets, mechanisms of actions, and their biomedical implications. BioFactors.

[23] Pakravan P., Kashanian S., Khodaei M.M., Harding F.J. (2013). Biochemical and pharmacological characterization of isatin and its derivatives: From structure to activity. Pharmacol. Rep..

[24] Evdokimov N.M., Magedov I.V., McBrayer D., Kornienko A. (2016). Isatin derivatives with activity against apoptosis-resistant cancer cells. Bioorg. Med. Chem. Lett..

[25] Chu W., Rothfuss J., Zhou D., MacH R.H. (2011). Synthesis and evaluation of isatin analogs as caspase-3 inhibitors: Introduction of a hydrophilic group increases potency in a whole cell assay. Bioorg. Med. Chem. Lett..

[26] Chinchar E., Makey K.L., Gibson J., Chen F., Cole S.A., Megason G.C., Vijayakumar S., Miele L., Gu J.W. (2014). Sunitinib significantly suppresses the proliferation, migration, apoptosis resistance, tumor angiogenesis and growth of triple-negative breast cancers but increases breast cancer stem cells. Vasc. Cell.

[27] Alkahtani H.M., Alanazi M.M., Aleanizy F.S., Alqahtani F.Y., Alhoshani A., Alanazi F.E., Almehizia A.A., Abdalla A.N., Alanazi M.G., El-Azab A.S. (2019). Synthesis, anticancer, apoptosis-inducing activities and EGFR and VEGFR2 assay mechanistic studies of 5,5-diphenylimidazolidine-2,4-dione derivatives: Molecular docking studies. Saudi Pharm. J..

[28] Nosol K., Romane K., Irobalieva R.N., Alam A., Kowal J., Fujita N., Locher K.P. (2020). Cryo-EM structures reveal distinct mechanisms of inhibition of the human multidrug transporter ABCB1. Proc. Natl. Acad. Sci. USA.

[29] Cooper M.A. (2003). Label-free screening of bio-molecular interactions. Anal. Bioanal. Chem..

[30] Caputo M., De Rosa M.C., Rescigno T., Zirpoli H., Vassallo A., De Tommasi N., Torino G., Tecce M.F. (2014). Binding of polyunsaturated fatty acids to LXRα and modulation of SREBP-1 interaction with a specific SCD1 promoter element. Cell Biochem. Funct..

[31] Malafronte N., Vassallo A., Dal Piaz F., Bader A., Braca A., De Tommasi N. (2012). Biflavonoids from Daphne linearifolia Hart. Phytochem. Lett..

[32] Dal Piaz F., Vera Saltos M.B., Franceschelli S., Forte G., Marzocco S., Tuccinardi T., Poli G., Nejad Ebrahimi S., Hamburger M., De Tommasi N. (2016). Drug Affinity Responsive Target Stability (DARTS) Identifies Laurifolioside as a New Clathrin Heavy Chain Modulator. J. Nat. Prod..

[33] Terracciano S., Chini M.G., Vaccaro M.C., Strocchia M., Foglia A., Vassallo A., Saturnino C., Riccio R., Bifulco G., Bruno I. (2016). Identification of the key structural elements of a dihydropyrimidinone core driving toward more potent Hsp90 C-terminal inhibitors. Chem. Commun..

[34] Schulte T.W., Akinaga S., Soga S., Sullivan W., Stensgard B., Toft D., Neckers L.M. (1998). Antibiotic radicicol binds to the N-terminal domain of Hsp90 and shares important biologic activities with geldanamycin. Cell Stress Chaperones.

[35] Guo W., Reigan P., Siegel D., Zirrolli J., Gustafson D., Ross D. (2005). Formation of 17-allylamino-demethoxygeldanamycin (17-AAG) hydroquinone by NAD(P)H:quinone oxidoreductase 1: Role of 17-AAG hydroquinone in heat shock protein 90 inhibition. Cancer Res..

[36] Camero C.M., Vassallo A., De Leo M., Temraz A., De Tommasi N., Braca A. (2018). Limonoids from Aphanamixis polystachya Leaves and Their Interaction with Hsp90. Planta Med..

[37] Amaral M., Kokh D.B., Bomke J., Wegener A., Buchstaller H.P., Eggenweiler H.M., Matias P., Sirrenberg C., Wade R.C., Frech M. (2017). Protein conformational flexibility modulates kinetics and thermodynamics of drug binding. Nat. Commun..

[38] Poli G., Lapillo M., Granchi C., Caciolla J., Mouawad N., Caligiuri I., Rizzolio F., Langer T., Minutolo F., Tuccinardi T. (2018). Binding investigation and preliminary optimisation of the 3-amino-1,2,4-triazin-5(2H)-one core for the development of new Fyn inhibitors. J. Enzym. Inhib. Med. Chem..

[39] Dal Piaz F., Ferro P., Vassallo A., Vasaturo M., Forte G., Chini M.G., Bifulco G., Tosco A., De Tommasi N. (2015). Identification and mechanism of action analysis of the new PARP-1 inhibitor 2″-hydroxygenkwanol A. Biochim. Biophys. Acta Gen. Subj..

[40] Domotor O., Tuccinardi T., Karcz D., Walsh M., Creaven B.S., Enyedy E.A. (2014). Interaction of anticancer reduced Schiff base coumarin derivatives with human serum albumin investigated by fluorescence quenching and molecular modeling. Bioorg. Chem..

[41] Milella L., Milazzo S., De Leo M., Vera Saltos M.B., Faraone I., Tuccinardi T., Lapillo M., De Tommasi N., Braca A. (2016). Alpha-Glucosidase and alpha-Amylase Inhibitors from *Arcytophyllum thymifolium*. J. Nat. Prod..

[42] Granchi C., Caligiuri I., Bertelli E., Poli G., Rizzolio F., Macchia M., Martinelli A., Minutolo F., Tuccinardi T. (2017). Development of terphenyl-2-methyloxazol-5(4H)-one derivatives as selective reversible MAGL inhibitors. J. Enzym. Inhib. Med. Chem..

[43] Vine K.L., Belfiore L., Jones L., Locke J.M., Wade S., Minaei E., Ranson M. (2016). N-alkylated isatins evade P-gp mediated efflux and retain potency in MDR cancer cell lines. Heliyon.

[44] Sevin M., Girodon F., Garrido C., de Thonel A. (2015). HSP90 and HSP70: Implication in Inflammation Processes and Therapeutic Approaches for Myeloproliferative Neoplasms. Mediat. Inflamm..

[45] Bentires-Alj M., Barbu V., Fillet M., Chariot A., Relic B., Jacobs N., Gielen J., Merville M.P., Bours V. (2003). NF-kappaB transcription factor induces drug resistance through MDR1 expression in cancer cells. Oncogene.

[46] Kim H.B., Lee S.H., Um J.H., Oh W.K., Kim D.W., Kang C.D., Kim S.H. (2015). Sensitization of multidrug-resistant human cancer cells to Hsp90 inhibitors by down-regulation of SIRT1. Oncotarget.

[47] Yin L., Yang Y., Zhu W., Xian Y., Han Z., Huang H., Peng L., Zhang K., Zhao Y. (2021). Heat Shock Protein 90 Triggers Multi-Drug Resistance of Ovarian Cancer via AKT/GSK3beta/beta-Catenin Signaling. Front. Oncol.

[48] Dinic J., Podolski-Renic A., Jovanovic M., Musso L., Tsakovska I., Pajeva I., Dallavalle S., Pesic M. (2019). Novel Heat Shock Protein 90 Inhibitors Suppress P-Glycoprotein Activity and Overcome Multidrug Resistance in Cancer Cells. Int. J. Mol. Sci..

[49] Bader A., Bkhaitan M.M., Abdalla A.N., Abdallah Q.M.A., Ali H.I., Sabbah D.A., Albadawi G., Abushaikha G.M. (2021). Design and Synthesis of 4-O-Podophyllotoxin Sulfamate Derivatives as Potential Cytotoxic Agents. Evid. Based Complement. Altern. Med..

[50] Al-Salem H.S., Arifuzzaman M., Alkahtani H.M., Abdaalla A.N., Issa I.S., Alqathama A., Albalawi F.S., Rahman A. (2020). A Series of Isatin-Hydrazones with Cytotoxic Activity and CDK2 Kinase Inhibitory Activity: A Potential Type II ATP Competitive Inhibitor. Molecules.

[51] Al Bratty M., Makeen H.A., Alhazmi H.A., Syame S.M., Abdalla A.N., Homeida H.E., Sultana S., Ahsan W., Khalid A. (2020). Phytochemical, Cytotoxic, and Antimicrobial Evaluation of the Fruits of Miswak Plant, *Salvadora persica* L.. J. Chem..

[52] Abdalla A.N., Abdallah M.E., Aslam A., Bader A., Vassallo A., Tommasi N., Malki W.H., Gouda A.M., Mukhtar M.H., El-Readi M.Z. (2020). Synergistic Anti Leukemia Effect of a Novel Hsp90 and a Pan Cyclin Dependent Kinase Inhibitors. Molecules.

[53] Abdalla A.N., Shaheen U., Abdallah Q.M.A., Flamini G., Bkhaitan M.M., Abdelhady M.I.S., Ascrizzi R., Bader A. (2020). Proapoptotic Activity of Achillea membranacea Essential Oil and Its Major Constituent 1,8-Cineole against A2780 Ovarian Cancer Cells. Molecules.

[54] Berman H.M., Westbrook J., Feng Z., Gilliland G., Bhat T.N., Weissig H., Shindyalov I.N., Bourne P.E. (2000). The Protein Data Bank. Nucleic Acids Res..

[55] Morris G.M., Huey R., Lindstrom W., Sanner M.F., Belew R.K., Goodsell D.S., Olson A.J. (2009). AutoDock4 and AutoDockTools4: Automated docking with selective receptor flexibility. J. Comput. Chem..

[56] Granchi C., Lapillo M., Glasmacher S., Bononi G., Licari C., Poli G., El Boustani M., Caligiuri I., Rizzolio F., Gertsch J. (2019). Optimization of a Benzoylpiperidine Class Identifies a Highly Potent and Selective Reversible Monoacylglycerol Lipase (MAGL) Inhibitor. J. Med. Chem..

[57] Roe D.R., Cheatham T.E. (2013). PTRAJ and CPPTRAJ: Software for Processing and Analysis of Molecular Dynamics Trajectory Data. J. Chem. Theory Comput..

[58] Poli G., Gelain A., Porta F., Asai A., Martinelli A., Tuccinardi T. (2016). Identification of a new STAT3 dimerization inhibitor through a pharmacophore-based virtual screening approach. J. Enzym. Inhib. Med. Chem..

[59] Eid S.Y., Althubiti M.A., Abdallah M.E., Wink M., El-Readi M.Z. (2020). The carotenoid fucoxanthin can sensitize multidrug resistant cancer cells to doxorubicin via induction of apoptosis, inhibition of multidrug resistance proteins and metabolic enzymes. Phytomedicine.

[60] Eid S.Y., El-Readi M.Z., Eldin E.E., Fatani S.H., Wink M. (2013). Influence of combinations of digitonin with selected phenolics, terpenoids, and alkaloids on the expression and activity of P-glycoprotein in leukaemia and colon cancer cells. Phytomedicine.

[61] Abdalla A.N., Qattan A., Malki W.H., Shahid I., Hossain M.A., Ahmed M. (2020). Significance of Targeting VEGFR-2 and Cyclin D1 in Luminal-A Breast Cancer. Molecules.

[62] Almasmoum H., Refaat B., Ghaith M.M., Almaimani R.A., Idris S., Ahmad J., Abdelghany A.H., BaSalamah M.A., El-Boshy M. (2019). Protective effect of Vitamin D3 against lead induced hepatotoxicity, oxidative stress, immunosuppressive and calcium homeostasis disorders in rat. Environ. Toxicol. Pharm..

[63] Abdallah M.E., El-Readi M.Z., Althubiti M.A., Almaimani R.A., Ismail A.M., Idris S., Refaat B., Almalki W.H., Babakr A.T., Mukhtar M.H. (2020). Tamoxifen and the PI3K Inhibitor: LY294002 Synergistically Induce Apoptosis and Cell Cycle Arrest in Breast Cancer MCF-7 Cells. Molecules.

